# Life-review interventions as psychotherapeutic techniques in psychotraumatology

**DOI:** 10.3402/ejpt.v4i0.19720

**Published:** 2013-05-20

**Authors:** Andreas Maercker, Rahel Bachem

**Affiliations:** Division of Psychopathology and Clinical Intervention, Department of Psychology, University of Zurich, Zurich, Switzerland

**Keywords:** Life-review, elderly, posttraumatic stress disorder, depression, case example

## Abstract

**Background:**

Life-review interventions (LRI) are psychotherapeutic techniques originally derived from gerontology, which can be distinguished from other biographical and reminiscing techniques. They have been systematically implemented and investigated not only in elderly clients with depression, cognitive decline, in oncology units and in hospices but also in adolescents with various mental problems. LRI are mainly based on the elaboration of the autobiographical memory as well as on personal identity consolidation. This bears the potential for the systematic introduction, use, and evaluation of LRI within the field of psychotraumatology.

**Method:**

This article gives a general overview and outlines a structured LRI by means of a case example of a World War II-traumatised patient. Other applications and implementations of LRI in psychotraumatology and other related areas are presented.

**Result:**

So far, only uncontrolled or controlled LRI case studies have been investigated with traumatized samples.

**Conclusion:**

The importance of further randomized controlled studies is emphasized.

Psychotraumatology has given rise to new inventions in the treatment of traumatic stress syndromes such as prolonged exposure, eye movement desensitization and reprocessing (EMDR) or narrative exposure. Recently, treatment methods for specific client or patient groups have been proposed, including phase-based treatment for complex PTSD (Cloitre, Cohen, & Koenen, 2006) and treatment of complicated grief (Shear, Boelen, & Neimeyer, 2011). Life-review interventions (LRI), which have originally been developed to treat psychological problems in the elderly, may be regarded as such a specialized treatment method. This article outlines their objective and rationale(s), and gives an outlook on their potential when applied for the treatment of trauma and stress-related disorders.^1^ Finally, a summary of the current state of research is provided.

Nearly all forms of psychotherapy have been developed by physicians or psychologists—whereas life-review techniques have been developed mainly by gerontologists and nursing scientists. LRI are psychotherapeutic techniques related to story-telling or biographical techniques and to reminiscing work (see Table 1) that have been applied in clinical gerontology for years and have been specifically developed for use within a psychotherapeutic work context (Haight & Webster, 1995).

**Table 1:** Definitions and fields of application of LRI, story-telling, and reminiscence work in comparison

Life-review interventions (structured form) (Haight & Haight, 2007)	Reminiscense work (Gibson, 2006)	Storytelling (Schiffrin, De Fina, & Nylund, 2010)
Group of psychotherapeutic techniques with a focus on integration of life history. Clients are asked to report their memories in detail, structured according to life-phases, e.g., childhood, adolescence, young, middle, and old adulthood. Breakages, contradictions, and failures as well as resources and strengths serve as reference points and content for cognitive restructuring. Life-review interventions showed their effectiveness for treating depressive disorders.	Intermediate demanding technique. It is conversation-based. Partly in one-on-one, partly in group conversations, clients are asked to tell about their memories in detail, ordered by life-phases, e.g., childhood, adolescence, young, middle, and old adulthood. Use of memorabilia is encouraged. No cognitive restructuring involved. This form is often applied with clients with chronic depression or early/mild forms of dementia.	This simpler form is oriented towards communication and interaction. Commonly used in elderly care facilities. It centres on gathering specific themes, e.g., festivities, holidays, popular former children's games or cooking recipes. Goals are general activation, sense of mastery, and rejoice/gratification by positive memories.

LRI are a group of treatment methods sharing similarities but comprising different variants. Especially unstructured forms of LRI that are common in psychodynamic treatment settings need to be differentiated from structured (manualized) interventions. In psychodynamics or psychoanalysis, these interventions merge more or less seamlessly into standard methods of particular therapeutic orientations, when used with elderly patients and when therapists have patients talk about their lives, starting with childhood (Blum & Tross, 2008). The focus of the following description will be a structured version of a LRI, specifically presented in its application for the treatment of PTSD.

## Previous applications

LRI have been developed in social gerontology for the work with people with Alzheimer's disease, other types of dementia and for mixed patient populations (e.g., depressive patients with cognitive decline) (Haight & Haight, 2007). A recent meta-analysis indicates that structured LRI show stronger intervention effects compared to reminiscence work or story-telling, but they are also highly demanding of the client's cognitive abilities (Pinquart & Forstmeier, 2012). Therefore, story-telling or reminiscing techniques are given preference when working with demented patients. Soon it was discovered that LRI effectiveness was specifically enhanced when treating patient groups with depression (unipolar depression, major depression, dysthymia; Pinquart, Duberstein, & Lyness, 2007; Pinquart & Forstmeier, 2012). They also proved to be favorable for the treatment of cancer patients, in hospices and in terminal care as well as in end-of-life care (Aylor & Grimes, 2008). LRI are further recommended in patients with profound medical diseases, such as stroke (ref).

LRI are not only effective when treating the elderly but they are also of great value for patients of other age groups—a fact that has achieved only limited notice in psychotherapy (Maercker & Forstmeier, 2012). Various evaluation projects and studies showed that even children and teenagers can benefit from therapeutic or social pedagogical forms of LRI if exploration of one's biography is indicated, for example, in foster children who have been separated from their biological parents or adolescents with a migration background (Morgenstern, 2011; Zalpour, Abedin, & Heidari, 2011). For middle adulthood, different treatment programs are available, especially if the person is physically sick. When examining the effect of LRI on depression in young and middle-aged adults compared to elder people, no differences in effect sizes could be detected, although only a few studies were conducted among these populations. It is noteworthy that especially middle aged patients suffering from chronic illnesses like cancer or AIDS, benefit from LRI to a large extent as—similar to elderly people—they are confronted with the finiteness of their life (Pinquart & Forstmeier, 2012).

## Application in psychotraumatology

The first report on the use of a reminiscence-based approach in treating Holocaust survivors in Israel was published in 1992 (Schindler, Spiegel, & Malachi, 1992). A decade later, Maercker (2002a) and Maercker and Mueller (2004) published several case reports using manualized LRI in elderly with chronic PTSD developed because of a World War II-related traumatization. Three case reports were examined using a controlled single case design (multiple-baseline), which showed stable treatment effects lasting for 3 months after treatment completion/cessation (Maercker, 2002a). The pre-treatment “Impact of Event-revised sum-scores” of 43 changed to 24 post-treatment and to 21 in the 3-months follow-up. The LRI manual used in these case studies has recently been updated and re-adapted for the treatment of Holocaust survivors who are now in their 80s and 90s (Forstmeier, Maercker, Van der Hal-van Raalte, & Auerbach, 2013).

A similar LRI manual, called Integrative Testimonal Therapy (ITT), was introduced by Knaevelsrud and colleagues (Böttche, Kuwert, & Knaevelsrud, 2012; Knaevelsrud, Böttche, & Kuwert, 2011) which in addition to the previous manual is based on “Testimony Therapy” with traumatized victims of organized violence (Cienfuegos & Monelli, 1983). The authors investigated its effects in a German population of elderly who were traumatized as children during World War II. Knaevelsrud et al. (2012) also report a randomized controlled trial of a web-based version of the ITT (“Lebenstagebuch”/Life diaries; see paragraph below on LRI in New Media) with significant improvements in the treatment group compared to the waiting list control group.

The two LRI manuals in psychotraumatology share some similarities with the Narrative Exposure Therapy (NET; Schauer, Neuner, & Elbert, 2005). Over the course of a small number of sessions (typically 3–4, in some cases up to 12), the client, together with the therapist, constructs a detailed and consistent narration of his or her biography. His or her traumatic experiences are written down and, depending on the willingness of the client, used for documentary purposes. Differences to LRI are the smaller number of sessions and the lack of self-reflexion or cognitive restructuring sessions in the last phase of treatment. Therefore, NET shows a stronger resemblance with reminiscence work compared to structured LRI (see Table 1). NET has proven to be efficient in samples of refugees across different countries, including Africa and Asia where on-site treatments have been performed (Robjant & Fazel, 2010).

So far, no evidence-based rule of decision can be deduced as to when and why the previously described LRI approaches should be chosen over prolonged exposure or other empirically validated treatments. The principle benefits for LRI use in work with elderly traumatized clients lies in the particularities of old age, which are better addressed in this technique compared to other procedures. Psychological gains and losses at this stage are assumed to be especially well addressed by this method and its underlying modes of action.

## Active ingredients of LRI

Investigation of active principles of LRI is still in its infancy. In recent years, life-span developmental psychology has discovered and accurately described many new relationships, most of which have not been applied yet to the treatment of mental disorders. The study of lifelong development of autobiographical memory, for example, demonstrated that there is a “reminiscence bump” with a surplus of memories between the age of 15 and 25 and that there are different levels of memory specificity (life period knowledge, area of life knowledge, event-specific knowledge) (Conway & Pleydell-Pearce, 2000). Furthermore, processes of life review in the strict sense can be distinguished from reflection on life. The former is aimed towards enriching memory processes whereas the latter is characterized by the formation of new insights and the questioning of old beliefs. It has been assumed that a therapeutic benefit is only achievable when a reflection-on-life component is added to life review (Staudinger, 2001). A number of findings contradict this assumption and suggest that even life review and remembering per se can have a mood-elevating effect (Pinquart & Forstmeier, 2012).

Memory research has investigated the so-called mood-congruency effect, stating that thoughts, judgments and free association performance run in accordance with the emotional state. Accordingly, more stressful episodes should be activated by autobiographical memory when the individual is in a state of negative emotionality. In the case of positive mood, more constructive memories are activated (Matt, Vázquez, & Campbell, 1992). In addition, other specificity effects were found in this context: depressive individuals remember stressful episodes in detail, whereas positive memories are presented only in a summary form—an effect that changes after successful completion depression therapy (Serrano, Latorre, Gatz, & Montanes, 2004). Overall, it seems that the mood-congruency effect becomes activated by a positive prevailing mood of a stable patient–therapist relationship where the therapist constantly reinforces the activation of positive memories and does not exclusively focus on negative or traumatic memories.

On a pragmatic level, three active principles of LRI can be described (see Maercker, 2002b):

1. Life Balance: The intervention promotes a balanced accounting of positive and negative memories (“ups and downs in life”). Positive memories (e.g., pleasant experiences, coping success, skills) should dominate over negative ones (e.g., failures, experiences of loss, trauma).
2. Find meaning: Negative experiences, including trauma, can be given a meaning. Even if trauma or death of a spouse remains a negative fact, the subjective experience of having been altered by the event in a positive sense, enables and supports a new extended view on one's own life.
3. Elaboration of memory, that is, greater detail of what is remembered actively. In depressed patients not only negative aspects are remembered, but also positive ones. In the case of trauma, memories should be elaborated and processed into a narrative.

## Life-review interventions and memory phenomena in PTSD patients

People with traumatic experiences and subsequent PTSD suffer from adverse effects on their ability to encode and integrate the traumatic experience in their autobiographical memory. Memories about the trauma itself are fragmented and disorganized when intentionally recalled (McNally, 2003 for a review). When unintentionally recalled, they appear in the form of negatively experienced, and uncontrollable intrusions (Brewin, Dalgleish, & Joseph, 1996; Ehlers & Clark, 2000). Traumatic memories are not isolated from other personal memories and can be connected to non-traumatic memories. Consequently, non-traumatic memories function as non-intentional triggers of trauma-relevant contents (Berntsen, 2001). Furthermore, this intentional recall of non-traumatic memories is less specific in traumatized persons than in non-traumatized persons (Williams et al., 2007).

LRI-therapy targets the revision of these processes—renewed and adaptive encoding of the fragmented traumatic memories enables the development of a narrative structure. Contents of the verbally accessible memory and associated contents of the situational accessible memory are connected to reduce involuntary recall of memories (intrusions). Moreover, LRI with PTSD-patients exceeds common trauma focusing by aiming for a broader and more adequate conception of the traumatic experience. The structured character of chronological recall and evaluation of life stages is also assumed to enhance the specificity of non-traumatic memories (Knaevelsrud, et al., 2012).

## Practical procedure

In the following, a typical approach will be outlined. For PTSD patients, LRI is divided into

- the initial phase, when the course of therapy and its relation to their main symptoms is explained to the patients/clients.
- the middle phase, when on the basis of an individually adapted plan, different stages of life are discussed in succession—in treatment of PTSD the phase of life in with the trauma happened is integrated here.
- the final phase, in which integrating and balancing therapeutic conversations and planning for the time after therapy (relapse prevention) are in focus.

In the *initial phase*, goals and actual processing are discussed with the patient. Depending on the level of education or comprehension of the patient, the reasons/rationales for the following process vary. In patients with good comprehension, all of the above suggested three active principles (life balance, meaning and elaboration) are discussed.

In patients with lower comprehension, more simple sentences are selected, for example, *Remembering childhood, often gives great pleasure and brings people in a good mood, which is useful for those who sometimes have problems. This can sometimes help with problems. I think that many people's lives are very interesting, and I would be glad if for the next hours you told me a few detailed stories you remember*. (In case the patient has been traumatized in early childhood, another anchor for positive memories has to be selected.)

The patient is informed that between 10 and 15 sessions are needed to discuss the important stages of their life. Furthermore, they are asked to bring appropriate personal memorabilia to each session (e.g., photos, letters, and diaries).

For some patients, it is extremely difficult to engage in a life review based on concrete experiences. In this case, guided imagination may be helpful so that first an exercise can be carried out (e.g., *Please imagine as vividly as possible that you are doing gardening or cleaning a room…*.). An initial resistance of telling memories (e.g., *I do not remember anything anymore, that's no use!*) may be due to depressive symptoms. With the help of general psychotherapeutic relationship work or by providing positive feedback for the first memories expressed, therapists can frequently overcome this resistance.

## Course of the middle phase

The process follows the succession of stages of life, with an individually set time frame, depending on the precedent anamnestic interview. Each period of life, from infancy to present age, will be thoroughly discussed in at least one session.

## Sample case of a life-review intervention for a traumatized patient

1. Childhood to schooldays I
2. Childhood to schooldays II: continued
3. School days to puberty
4. Youth to career start
  X. Traumatic experience (is placed before stage of life in which the event happened)
5. Adulthood I: working life, partnerships, until first child birth
6. Adulthood II: children from birth until moving out of family
7. Adulthood III: from age 50 to retirement
8. Retirement age to present

In the subsequent case study, two sessions for early childhood were set, because it is vital that enough LRI sessions (i.e., 3–4) have taken place before reviewing the trauma in adolescence (see details below). In this specific case, only one session was set for the time of “reminiscence bump” (see above) of 15–25 years (youth to career start) —more sessions can be implemented at this point for other cases.

The discussion of life cycle begins with childhood, such as *Today we will start talking about your life. Let us start more or less at the beginning. It is best to proceed chronologically, recounting the earliest memories first. At the moment, it is not so important how far we get with remembering. What are some of your earliest memories?* It is not only important that the patient describes the memories but that reflection over these memories is stimulated (*What did this mean to you then?*).

Questions about the life stage of adulthood, are for example, *How were you then? What did you value? What was important to you? What were your strengths? Did you enjoy your work? How important was this activity for you?*

## How complete must a life-review be?

It is not possible to conduct an extensive review within 5–8 sessions, neither is it necessary in order to meet the objectives of LRI. However, if the therapist notices large time gaps or missed issues (such as the negative emotional connotations), a more in-depth exploration of these periods is indicated (Maercker & Forstmeier, 2012). Alternatively, a list of topics that should be addressed can be presented, including (Haight & Haight, 2007) origin of family, school and education, perception of the cohort, sexual development, partnerships and marriages, children, professional biography, perceptions of ethnicity, gender and social class, body image and physical changes, religious and spiritual development, world view, experiences with death, and view of the future (e.g., *How much time is left? What are the things to be done?*). The therapist can approach the gaps with questions (e.g., *You tend to portray your mistakes, let's talk a little about your success! Now I know much about your first job, tell me something about your subsequent job*).

## Inclusion of the traumatic experience

In particular, elderly patients often find it difficult to talk about their traumatic experiences, especially if they suffer from a full-blown PTSD and when avoidance of thoughts and feelings associated with the trauma is fully developed (Maercker, 2003). In these cases, the gradual nature of LRI is a good way to encourage patients to talk about these experiences. In earlier stages of the therapy, they get familiar with the process and thus experience that memories can be controlled.

If the patient has experienced a trauma or—as it is the case of complicated grief—experienced the loss of an important person, a separate session is reserved to address this issue. The discussion takes place before the session on the phase of life in which the trauma happened, which is of special importance. The therapist expresses his/her empathy and explains that he knows how difficult it can be for the patient to deal with the terrible experience in full detail. At first, the narrative of the patient is not interrupted by questions for positive aspects (e.g., their own coping). This can be done afterwards or in the subsequent session with a focus on integration of life balance. The existential severity of the trauma is appreciated by the therapist (*That must have been a most terrible time for you*). Other elements of trauma narration prompt questions to go into more detail, personal thoughts and feelings, as it is important not to rush through these memories. However, in contrast to prolonged or narrative exposure procedures, the patient is not prompted to focus on sensory details if he or she does not bring them up spontaneously.

At the end of the session, the therapist asks for positive changes related to overcoming the trauma (e.g., *Have you noticed that you have learned something positive from this experience of life?*). In case of denial the theme of drawing a positive conclusion will be addressed at this point, *Did you find a conclusion for yourself? What does it look like or could look like?* It is essential to continue discussing the subsequent stages of life in the following sessions, since this implicitly supports a key purpose of life review *(The trauma is only one part of life*).

## Conclusion of therapy

In the final sessions, when experiences of individual stages of life are integrated and assessed—usually several times—, the following questions can be asked, *We have been talking about your life for a while. However, why don't you now give an account of your personal development, of what you have learned in life? What would you describe as the three most important things in your life? Why? What would you change, do better or leave unchanged? What are now the most important things in your life?*

These sessions work preferentially with cognitive restructuring techniques to overcome the remaining dysfunctional thoughts (e.g., *The trauma damaged parts of my personality forever*) or to positively reframe the patients’ experiences (e.g., *I lived my life intensely and experienced history closely*.). These sessions can extend therapeutic discussions of post-traumatic growth issues (Zoellner & Maercker, 2006).

As a supplementary therapeutic method, the patient may be instructed to write their biography during or after the treatment. This can be done in chronological order or by incorporating selected stages only. The biography should not include solely facts, but also the associated feelings at the time, as well as at present.

In some cases, it is advisable to apply other psychotherapeutic techniques after LRI. This may be a behavioural therapy to build up pleasant activities, or other forms of therapy such as cognitive therapy.

## Case study

Our psychotherapeutic center has reported and described LRI therapies in elderly patients with PTSD in the German language before (Maercker & Mueller, 2004).

*A 66-year-old woman noticed for years that she was touched strongly by the theme of war victims. Also, the war-related nightmares which she had been having more or less pronounced for long periods of her life had intensified in recent years*.

*At the age of six, she witnessed a heavy bombardment in an Austrian town. As her mother was not in the apartment at the time of the attack, the patient had to take care of her three younger siblings on her own. As another traumatic experience, she stated that she had suffered from typhoid fever at the age of 22 and the doctor had told her that she would not live very long. A current problem was that she was afraid of injury, since a large blood vessel runs irregularly through her knee and a violation could lead to massive bleeding. By means of structured diagnostic interviews, delayed PTSD, somatoform disorder and sleep disorder (insomnia) were diagnosed*.

*The life-review therapy began with a single session on pre-traumatic childhood, which she described as happy. Already in the second session, the trauma of the bombing was discussed. First, she described the events in a very summary form and she went into more detail only on request. The courage she had proved in protecting her younger siblings was appreciated in detail and confirmed. (Note: Considering the current state of knowledge we would no longer recommend to discuss pre-traumatic childhood for only one hour; reasons see above)*.

*In the third session, the typhoid fever and the patient's fear that she would die at age 22 were discussed. She was enabled to express her grief that years of her life had been taken from her because of the war. When asked about positive aspects in this phase of life, she replied that she had then begun to plan her life and to live consciously*.

*In the therapeutic conversations, the patient stated repeatedly that she had been thinking about writing a book on her traumatic experiences in war for a long time. Although the traumatic experiences have been very relevant in every phase of life, she had been delaying writing the book again and again for 30 years already. Instead, she took part in various charitable activities that she initiated*.

*In the course of therapy, rapid improvement was achieved. In particular, the involuntary stressful memories (intrusions) decreased. The psychological examination of her PTSD symptoms before, during and after treatment showed a sustained reduction of the values (Maercker,* 2002a*). Her difficulties of falling asleep and sleeping through the night did not improve, however. The patient stated that a reason for the continuing insomnia could be the heavy burden of her voluntary work*.

*For this patient, it was important that her reports of overcoming the traumatic experiences, her motivation to write a book about them and her present and former honorary posts were confirmed by the therapist*.

## Life-review interventions and the New Media

In an effort to provide new relevant client groups with LRI, various New Media applications such as the internet, e-mail or computer software have been developed (Preschl, Wagner, Forstmeier, & Maercker, 2011). These technologies allow us to include so-far under-served populations with psychological needs. New Media offer low-threshold access to qualified psychological interventions.

### LRI-websites

One way of providing low-threshold offers of LRI to a wide audience are websites, which enable users to write down and reflect on their personal life story without the support of a therapist, but guided by predetermined questions. A well-established website of this kind is www.lifebio.com, where the biographical text can be enriched with photos and shared with relatives—online as well as in printed form (Troy, 2006). Evaluations of this program are on-going.

### LRI computer supplements for face-to-face therapy

A second way of transferring LRI to the New Media is to include LRI modules in specific therapeutic software, similar to how it has been done in the “Butler-System” (Botella et al., 2009). This system has been translated, introduced and validated in our outpatient clinic and laboratory to be applied with elderly persons seeking psychotherapeutic or psychotraumatological support (Preschl et al., 2012). In the “Butler-System”, patients have the opportunity to communicate with their therapist via the Butler-platform and use texts, photos or music to personalize their multimedia life-reviews. Basically, they write down life episodes into their “lifebook” with the support of the therapist who encourages them to express their feelings and thoughts as they were at the time of the experiences. Subsequently, clients were encouraged to add suitable photos or music pieces. The user interface of the Butler-System can be adjusted to the sensory and cognitive abilities of the user and is therefore also applicable to elderly people.

Preschl and colleagues (Preschl et al., 2012) used the life-review module of the Butler-System as a supplement to traditional face-to-face LRI-therapy in a population of elderly adults over 65, with elevated depression scores and some extend of stressful life experiences but not “classic” PTSD diagnoses. Patients were randomly assigned to an intervention group and a waiting-list control group and completed a 6-week treatment with 12 sessions. With the current Butler-protocol, clients are not asked to report on their traumatic events in particular, however, they could select them when they were asked for their most important memories. Results of the present depression module showed a large effect from pre- to post-treatment as well as to a 3-month follow-up. Furthermore, well-being increased and obsessive reminiscence decreased, the latter indicating that intrusive memory processes constitute an effective treatment goal (Preschl et al., 2012).

### Online-treatment

As mentioned above, Knaevelsrud et al. (2012) have used the LRI method of “life-diaries” on the Internet (www.lebenstagebuch.de). Their treatment services are aimed at people over 65 who are currently suffering from psychological long-term consequences of traumatic experiences during, and shortly after, World War II.

Clients and their constantly assigned e-therapists communicate exclusively via the internet, and in exceptional cases via post or fax. The e-therapists follow a specified treatment manual deriving both from the LRI approach and online psychotherapy of PTSD (Knaevelsrud & Maercker, 2007). It consists components, such as (1) Life review: During this stage, life phases are recalled from childhood up to today in seven sessions of writing and with the use of memory prompts; (2) Self-confrontation: In this stage, two texts are written with a focus on the most stressful traumatic event(s) as detailed as possible; (3) Cognitive restructuring and farewell: In this stage, two texts are written describing experiences and learned lessons from the clients current view.

The e-therapists interact with their clients after one or two texts, by sending a personal response to the client as well as instructions for the following texts. All participating therapists are extensively trained in the application of this therapeutic approach (see Knaevelsrud et al., 2012).

## Evidence base of LRI

To date, no randomized controlled trial (RCT) on LRI has been conducted with traumatized samples. However, a large number of RCT have proven that the highly structured LRI is a very effective treatment against depressive symptoms. A previous meta-analysis found nine RCTs with LRI in which 520 patients were treated. The mean effect size of LRI was 0.92, which can be considered a very good therapeutic effect (Bohlmeijer, Smit, & Cuijpers, 2003).

In the most extensive recent meta-analysis (including 128 studies) conducted by Pinquart and Forstmeier (2012), these results were replicated. The mean effect size of structured LRI, including 14 RCT, was 1.28, which represents an improvement of symptoms in about 77% of patients and can be described as a very good therapeutic effect. Furthermore, the results correspond or exceed treatment effects with psychotropic drugs and other psychotherapeutic techniques. As summarized above, less structured story-telling had a smaller effect size of 0.52, whereas reminiscing work showed a medium effect size of 0.31 on depressive symptoms. The greater effect sizes of more structured LRI should be put into perspective by the fact that people attending structured LRI usually have higher levels of depression at baseline and consequently, a larger capacity for improvement than volunteering individuals for reminiscence work and other less structured forms of intervention.

While most meta-analysis mainly investigated depressive symptoms, Pinquart and Forstmeier (2012) included a variety of other outcome measures delineated from life-span or existential psychology (e.g., Erikson & Erikson, 1998; Frankl, 1988). The effects ranged from moderate-to-high (*g* between 0.40 and 0.64) to small (*g* between 0.24 and 0.39): Ego-integrity (*g*=0.64), purpose in life (*g*=0.48), death preparation (*g*=0.40), mastery (*g*=0.40), mental health (*g*=0.33), positive well-being (*g*=0.33), social integration (*g*=0.31) and cognitive performance (*g*=0.23) were significantly improved when LRI or reminiscing techniques and story-telling were applied.

Pinquart and Forstmeier (2012) provide evidence that persons suffering from chronic and life-threatening illnesses, such as AIDS or cancer, benefit more from reminiscence interventions than healthy individuals. This effect could be due to the limited life-expectancy of these patients and the increased sensitivity to achieve a sense of ego-integrity.

## Discussion and outlook

To date, only single case studies reporting high effects are available for LRI-effects on PTSD (see above). There is a need for quantitative studies examining the effect of LRI on PTSD-symptoms as well as other aspects of mental health and well-being. Many of the existing studies evaluating the connection between LRI and depression do not include follow-up measurements and consequently little is known about the long-term effects of LRI.

Future research could examine whether certain groups of people, for example, individuals with unresolved biographical conflicts, profit to a higher degree from LRI compared to individuals with little or no unresolved biographical conflicts (Pinquart & Forstmeier, 2012). Furthermore, studies investigating the effect of LRI in young people and middle aged adults need to be conducted, as preliminary results from meta-analyses suggest a decrease in depressive symptoms across all age groups. Also, different age groups should be evaluated when examining LRI in PTSD patients. Even though in terms of effect sizes, LRI are comparable to other psychotherapeutic treatments, only a few studies exist, which directly compare the effectiveness of LRI and psychotherapeutic techniques such as cognitive behavioral therapy or the treatment with psychotropic drugs. Finally, LRI can be applied in the treatment of complicated grief symptoms in order to elaborate central themes in a person's life that are not connected to the deceased person. Empirical studies using LRI in patients with complicated grief are still missing, even though the loss of loved ones is one of the most frequent stressful experiences in old age.

The applicability of LRI as a preventive method for depressive disorders in older adults was demonstrated by Pot et al. (2010) who evaluated a life-review course called “Looking for Meaning” which covered various topics related to the course of life during 12 sessions which helped clients to link the past to the present. The minimal intervention comparison group was shown an educational video on successful aging. At post-test, the experimental group reported less depressive symptoms and a higher level of control over one's life than the control group. A remarkable between-group effect size of *d*=0.58 underscores the potential of LRI as a preventive technique for elderly people with an increased risk for depression.

LRI have a positive effect on various aspects of human experiencing and behavior: when structured LRI are applied in a psychotherapeutic setting with depressive patients, considerable therapeutic effects can be achieved. Not only do the depressive symptoms improve but also a range of other variables such as well-being and ego integrity. As outlined, we regard the potential for a broader introduction of LRI into psychotraumatology a promising setting for further advancement and differentiation of PTSD treatments.

## Footnotes

1 This article is the written version of the invited presentation at the 12th European Conference on Traumatic Stress 2011 in Vienna. It is partly based on a German advanced education material: Maercker, A. (2009). Lebensrueckblicksinterventionen als wirksame Alterspsychotherapietechniken. *Aerztliche Psychotherapie, 4*, 10–16
